# Perspectives of people in Mali toward genetically-modified mosquitoes for malaria control

**DOI:** 10.1186/1475-2875-9-128

**Published:** 2010-05-14

**Authors:** John M Marshall, Mahamoudou B Touré, Mohamed M Traore, Shannon Famenini, Charles E Taylor

**Affiliations:** 1 Division of Biology, California Institute of Technology, Pasadena, California, USA; 2 Malaria Research and Training Centre, University of Bamako, Bamako, Mali; 3 Department of Ecology and Evolutionary Biology, University of California at Los Angeles, Los Angeles, California, USA; 4 Center for Society and Genetics, University of California at Los Angeles, Los Angeles, California, USA

## Abstract

**Background:**

Genetically-modified (GM) mosquitoes have been proposed as part of an integrated vector control strategy for malaria control. Public acceptance is essential prior to field trials, particularly since mosquitoes are a vector of human disease and genetically modified organisms (GMOs) face strong scepticism in developed and developing nations. Despite this, in sub-Saharan Africa, where the GM mosquito effort is primarily directed, very little data is available on perspectives to GMOs. Here, results are presented of a qualitative survey of public attitudes to GM mosquitoes for malaria control in rural and urban areas of Mali, West Africa between the months of October 2008 and June 2009.

**Methods:**

The sample consisted of 80 individuals - 30 living in rural communities, 30 living in urban suburbs of Bamako, and 20 Western-trained and traditional health professionals working in Bamako and Bandiagara. Questions were asked about the cause of malaria, heredity and selective breeding. This led to questions about genetic alterations, and acceptable conditions for a release of pest-resistant GM corn and malaria-refractory GM mosquitoes. Finally, participants were asked about the decision-making process in their community. Interviews were transcribed and responses were categorized according to general themes.

**Results:**

Most participants cited mosquitoes as one of several causes of malaria. The concept of the gene was not widely understood; however selective breeding was understood, allowing limited communication of the concept of genetic modification. Participants were open to a release of pest-resistant GM corn, often wanting to conduct a trial themselves. The concept of a trial was reapplied to GM mosquitoes, although less frequently. Participants wanted to see evidence that GM mosquitoes can reduce malaria prevalence without negative consequences for human health and the environment. For several participants, a mosquito control programme was preferred; however a transgenic release that satisfied certain requirements was usually acceptable.

**Conclusions:**

Although there were some dissenters, the majority of participants were pragmatic towards a release of GM mosquitoes. An array of social and cultural issues associated with malaria, mosquitoes and genetic engineering became apparent. If these can be successfully addressed, then social acceptance among the populations surveyed seems promising.

## Background

Mosquito-borne diseases such as malaria and dengue fever continue to pose a major health problem through much of the world. Malaria alone causes over one million fatalities every year, the vast majority of them being children under five years old in sub-Saharan Africa [1,2]. Malaria control programmes in Africa have had very limited success throughout the years [3,4], and in the absence of a single effective strategy there has been increasing interest in integrated vector control strategies [5] and the distribution of anti-malarial drugs. The former would incorporate indoor residual spraying with insecticides, insecticide-treated bed nets and removal of mosquito breeding sites. Recently, it has been suggested that genetically-modified (GM) mosquitoes unable to transmit the malaria parasite be considered as a component in such a strategy [6]. In the case of malaria, a gene drive system would likely be used to drive refractory genes through mosquito populations [7], essentially replacing the population of susceptible vectors.

Public acceptance is essential prior to field trials of transgenic mosquitoes, particularly since mosquitoes are a vector of human disease and genetically modified organisms (GMOs) face strong scepticism in both developed and developing nations [8,9]. As stated by Macer [10], "*There is a need to engage the community and have two-way communication between researchers, policy makers and local communities in order to find whether each particular community will want to have a field trial, the nature of the concerns they have, and the ways that can be designed to involve communities as partners in trials*." The call to involve community members and exchange information with them that will ultimately contribute to how the disease control strategy is implemented has been made by several researchers [11-13]. This is particularly important in the early stages when the field trial protocol is being developed.

The protocol for contained field trials where transgenic mosquitoes may eventually be released is already being discussed [13,14]. Additionally, several surveys have been conducted on public attitudes to GMOs in Western nations [15-17], and at least one has asked people their views on GM mosquitoes for disease control [18]. Despite this, in sub-Saharan Africa where the GM mosquito effort is primarily directed, there is significantly less data available on public perspectives to GMOs, and data on perspectives to GM mosquitoes is virtually non-existent. To address this knowledge gap, a qualitative survey was conducted of public attitudes to biotechnology in rural and urban areas of Mali, West Africa between the months of October 2008 and June 2009. Here, perspectives of community members to the use of GM mosquitoes for malaria control are recounted along with the ethical, social and cultural issues that the project raises for them and for the communities to which they belong.

## Methods

### Study population

Mali is home to several different ethnic groups, including the Bambara, Dogon, Peul, Songhai and Tuareg. Malaria is prevalent in sub-Saharan Mali and the country is the site of extensive research on the ecology of malaria vectors of relevance to the GM mosquito project [19]. The literacy rate and level of education are low [20]. Education levels are lowest in rural areas and among females, who have a significantly lower rate of school enrolment than males.

The sample consisted of 80 individuals - 30 living in rural communities, 30 living in urban suburbs of Bamako, and 20 Western-trained and traditional health professionals working in Bamako and Bandiagara. For the rural sample, ten people were interviewed from each of three villages - Banambani and Tienfala in the region of Koulikoro, and Kaporo-na in the region of Mopti. Each village had a population size between 600 and 1000 people. Most rural participants were farmers and community decision-makers; while others identified themselves as students, teachers, artists and cooks. For the urban sample, ten people were interviewed from each of three suburbs of Bamako - Sabalibougou, Baco Djikoroni and Sans Fil. Urban participants belonged to a variety of professions - some sold food and appliances; others were teachers, hairdressers, cooks, taxi drivers, mechanics, plumbers and students. For the sample of health professionals, ten Western-trained doctors and scientists and ten traditional herbal and spiritual medicine practitioners were interviewed. Seven were doctors and three were researchers. All of them had attended university, and seven had completed a medical degree and/or PhD. The sample of traditional medicine practitioners either prepared or sold herbal remedies or practiced spiritual medicine for illnesses that were unresponsive to herbal remedies.

With the exception of Western-trained doctors and scientists, almost half of the participants had received little or no formal education. A few had attended university, while the remainder had attended Arabic or public schools. Participants were between the ages of 20 and 88 and, in all samples, 60-70% were male. Most participants identified themselves as Muslim, although there were a few Christians and one atheist. For more detailed demographic information, refer to Additional file 1.

### Research protocol

The interviews were conducted by an interviewer and a translator, sometimes in the company of another doctor or scientist. The translator - always a Malian national - posed a series of questions following a semi-structured questionnaire with limited input from the interviewer. The participant's responses were translated into English by the translator and transcribed by the interviewer. A tape recording of each meeting was made to check for errors.

The procedure for selecting participants varied according to the sample. For the rural sample, a local medical anthropologist at the Malaria Research and Training Center in Bamako suggested three villages - one with a history of entomological research (Banambani, a small Bambara village) [21]; and two without a history of medical research, one predominantly Bambara in ethnicity (Tienfala) and the other predominantly Dogon (Kaporo-na). The interview team then visited the chief of each village, explained the purpose of the survey and expressed a desire to interview both males and females representing a variety of age groups and social statuses. In Kaporo-na, the survey was explained to the mayor in lieu of the chief. In Banambani and Tienfala, the chief agreed to meet with the village elders later that day to coordinate the visit and invited the interview team back to the village the next day. Upon their return, the chief offered to be the first survey participant, which was likely part of the approval procedure. Following the interview, he assigned the interview team a young male guide who took them from one participant to the next. The chief and elders had already chosen the participants and provided a diverse range of men and women of various ages, although with a bias towards decision-makers. In Kaporo-na, the mayor offered to be interviewed the same day. He then arranged for the interview team to speak to the village decision-makers who were invited to be interviewed in the village chief's courtyard.

For the urban sample, a list of three suburbs was suggested by a local medical anthropologist and translator. These were selected for their spatial distribution and to capture a variety of socio-economic demographics. Upon visiting each suburb, households were randomly selected with the goal of achieving a good spatial spread. The interview team were usually welcomed into these households and introduced to the most senior person present. Sometimes they were invited back at a time when the head of the household would be present. The interview team explained the purpose of the survey to this individual and expressed a desire to interview both males and females representing a variety of age groups. The desire to interview females was emphasized to achieve a more balanced participant gender ratio. The senior household member usually accepted to take part in the survey and arranged for the interview team to speak to one of the household members. One person was interviewed from each household.

For the health professional sample, doctors, scientists and traditional healers were suggested by head scientists at the Malaria Research and Training Center in Bamako. Women and men from a variety of institutions were sought and, for doctors and scientists, eligibility was restricted to individuals who were not themselves involved in the GM mosquito project. Emphasis was placed on women during the selection procedure due to the fact that doctors, scientists and traditional healers in Mali are predominantly male. The interview team then visited these individuals, explained the purpose of the survey to them and asked whether they would like to participate. Most agreed to take part in the survey. Unlike other samples, many doctors and scientists preferred to write responses to survey questions on a form. The interview team returned to collect these from them.

Participants were offered a confidential setting to respond to survey questions; however this was seen as unnecessary by interviewees. Rural and urban participants appeared comfortable in a common setting with friends and relatives surrounding them and entering into discussions at times. Traditional healers appeared comfortable in their place of work.

### Survey questions

Interviews lasted 45 minutes on average. The survey was anonymous, however demographic details were requested. Questions were first asked about the cause of malaria, the intrinsic value of the environment, and cultural views towards living creatures. Participants were asked about resemblances between parents and offspring and whether their community takes advantage of this to selectively breed more desirable animals, vegetables or fruits. In this context, genetic alteration was described as "a faster way to develop more desirable animals, fruits and vegetables, but that this method could lead to unknown consequences for the environment." Participants were asked about the acceptability of growing GMOs in their community and any concerns they have regarding this technology.

The example of pest-resistant GM corn was then raised with the rural sample. "Imagine that an organization from a foreign country gifts you a more desirable fruit or vegetable that has been produced by this method [genetic engineering]; for example, a corn that is resistant to insects," participants were told. "A representative from the organization tells you that the crop has no negative consequences for the environment. Under what circumstances would you trust the representative and their statement that there are no negative consequences for the environment?" Participants were then asked under what conditions, if any, it would be acceptable for the GM crop to be grown in their community. The urban sample and health professionals were also asked their opinions on several applications of modern biotechnology, including GM food and insulin-producing GM bacteria.

Participants were then asked their opinions on GM mosquitoes for disease control. "Now imagine that an organization from a foreign country claims that they can provide you with a mosquito that has been produced by this method [genetic engineering] that is able to reduce the burden of malaria in your community," participants were told. "A representative from the United Nations tells you that the mosquito has no known negative consequences for your community or the environment. They do tell you, however, that there could be possible unknown negative consequences. How much trust would you have in the claim of the foreign organization that they can reduce the burden of malaria by releasing these mosquitoes?" Participants were also asked for their questions and concerns regarding this project and under what conditions, if any, they would consider it acceptable for the foreign organization to release GM mosquitoes into their community.

Finally, participants were asked how decisions are arrived at in their community when there are implications for all community-members, and who is involved in this process. Participants who gave detailed responses to these questions were asked whether they knew of any realistic ways in which their community can contribute to decisions made on a larger scale, and whether their community can object to decisions made on a national level. The urban sample and health professionals were asked which organizations they consider to be best-placed to regulate modern biotechnology, and whether there is a presence of stories on biotechnology in the Malian media. Informal conversation on these issues would sometimes continue for up to an hour following the interview.

Each interview was transcribed in its entirety, including any subsequent discussion. General themes were identified and responses categorized by a team of three researchers at the University of California at Los Angeles. Implications for a transgenic release were determined from this analysis.

### Ethical clearance

The public attitude survey was reviewed and approved by the institutional review boards of the Malaria Research and Training Centre (Bamako, Mali) and the University of California at Los Angeles (Los Angeles, CA, UCLA IRB #G08-04-081-01). Informed consent was obtained from all participants after the study was explained in the local language (either Bambara, Dogon or French). The survey was anonymous, and so consent was denoted by a research information sheet which was signed by the interviewer and translator to assure that the information had been effectively communicated. A recruitment letter explaining the survey and providing contact details for the interviewers was also provided to participants. Documentation is available in Additional file 2.

No payment was provided in return for participation in the survey; however compensation was given to village chiefs in the form of kola nuts and a soccer ball for the community. It was explained in the research information sheet that individual subjects would not directly benefit from participation in the research although "the results of the research may help researchers and decision-makers understand the perspectives of their community to the use of GM mosquitoes to control diseases such as malaria."

## Results

### Perceived causes of malaria

Over 80% of participants cited mosquitoes as at least one of the main causes of malaria and the majority of participants cited mosquitoes as the sole main cause of malaria. As one participant explained: "*When a mosquito bites, it can then bite another person and carry blood from the first person to the second person. This is how malaria is transmitted*" (Excerpt from an interview with a 70-year-old woman in Banambani). In urban areas, the presence of mosquitoes was often blamed on environmental dirtiness.

Other main causes of malaria that were cited include poor hygiene, tiredness, working too hard in the sun, dehydration, cold weather, not wearing enough clothes after a swim, oily foods such as peanuts, and sweet foods such as sugar, mangoes and plantains. These other causes were largely independent of mosquitoes, but they were usually cited in conjunction with mosquitoes (Table 1). The following explanation was typical: "*They say it's mosquitoes; but it's not only mosquitoes... During the months without mosquitoes, malaria is caused by foods such as peanuts and potatoes*" (Excerpt from an interview with a 52-year-old man in Kaporo-na). Doctors and scientists were well aware of the role of mosquitoes, often citing the scientific name of the most common malaria vector, *Anopheles gambiae*.

**Table 1:** Perceived causes of malaria.

Perceived causes of malaria	Rural areas*	Urban areas*	Doctors & scientists*	Traditional healers*	Total*
Mosquitoes	20	20	9	5	54

Mosquitoes & other causes	5	7	-	2	14

Nothing to do with mosquitoes	5	3	-	3	11

Dirtiness (attracts mosquitoes)	3	13	2	-	18

Foods (sweet foods, oily foods)	4	5	-	5	14

Dirtiness (general dirtiness, dirty water, dirty food)	3	5	-	1	9

Weather (cold weather, rain, wind, working in the sun)	4	2	-	2	8

Other insects (flies, other blood-feeding insects)	1	1	-	1	3

Rain, standing water (attracts mosquitoes)	1	2	-	-	3

Neem tree (attracts mosquitoes)	-	1	-	-	1

Malnutrition	1	-	-	-	1

Constipation	-	1	-	-	1

*Number of interviewees

### Views towards mosquitoes and other living creatures

Indigenous cultures often have valued species that they treat with heightened respect; however mosquitoes were singled out by participants as a nuisance, and killing them was almost unanimously acceptable. In reference to mosquitoes and flies, participants noted: "*If there is a solution to kill them, it will be better for our health; but creatures like dogs and cats, we should protect them*" (Excerpt from an interview with a 48-year-old woman in Baco Djikoroni). Only two participants voiced concerns over mosquitoes being killed; however both favoured an approach to malaria control in which the anti-malarial trait spreads into the mosquito population. One urban participant made reference to insects as possible objects of sorcery, highlighting the importance of studying local beliefs and avoiding negative cultural interpretations when preparing for a release of modified insects.

### Understanding of heredity

Most villages in Mali are primarily agricultural so their inhabitants are very familiar with the practice of selecting the most desirable animals and crops and breeding them to obtain more desirable offspring. Rural participants cited experience of selective breeding with animals such as chicken, goats, cows, sheep and pigs. As one participant explained: "*If you have a strong male goat, then you can take it to your farm to mate with female goats. They can then have strong babies*" (Excerpt from an interview with a 28-year-old man in Kaporo-na). They also cited practicing selective breeding with cereal crops and fruit trees: "*We take advantage of this resemblance to breed better varieties of many species, including orange and mango trees*" (Excerpt from an interview with a 48-year-old woman in Banambani). This implies a strong practical knowledge of heredity.

Participants in both rural and urban areas cited God and/or sharing the same blood as the main reasons why offspring tend to resemble their parents. Blood was often seen as the hereditary material; while God was seen as the source of variability. Participants noted: "*You can have some children who have darker skin, and some who have lighter skin. This happens because of God*" (Excerpt from an interview with an 88-year-old man in Tienfala). Participants offered several other causes of heredity as listed in Table 2. Only a very few educated people made reference to science and genetics - two from rural and four from urban areas. Toward the end of the survey, a local medical anthropologist suggested that the interview team start using the Bambara word "sikisè" - a concatenation of the word "siya", or race/ethnicity/species, with "kisè", or grain - to denote the concept of a gene. None of the ten urban participants questioned made any connection between the term sikisè and heredity; however two bystanders did. As one bystander described: "*This is the reason why you are what you are. If you're an animal, it's because of the sikisè. If you're a human, it's because of the sikisè*" (Excerpt from field notes during an interview with a resident in Sans Fil). It is important to be familiar with indigenous analogues of the gene; however the interview team found that it was most helpful to describe the gene by referring to its role in determining traits.

**Table 2:** Understanding of heredity.

Perceived reasons why offspring tend to resemble their parents	Rural areas*	Urban areas*	Total*
God	7	8	15

Blood	7	8	15

Blood and God	-	4	4

Lineage, race, ethnicity	2	6	8

Affection, relationship	7	-	7

Genes	1	-	1

Genes and God	-	2	2

Science and God	1	1	2

Don't know	6	2	8

* Number of interviewees

### Perception of genetic engineering

At the time of the survey there had been no commercial release of GM crops in Africa outside of South Africa. A commercial release of GM cotton had been approved in Burkina Faso; but commercial releases of GM crops had not been approved in any other African country, including Mali. Two urban participants were familiar with the GM cotton controversy in neighbouring Burkina Faso. As one of them summarized: "*The politicians in Burkina Faso made a decision about growing GM cotton without consulting the population*" (Excerpt from an interview with a 60-year-old woman in Sans Fil). Another four urban participants were aware of the problems of terminator seed technology, having heard these stories on television and radio. There was also an awareness of GM corn and rice being harvested abroad.

Mangoes were often identified as genetically modified. A few participants realized that these were the product of grafting by local agriculturalists: "*You can graft two varieties of mangoes together to get bigger mangoes*" (Excerpt from an interview with a 52-year-old man in Sans Fil). Several participants judged GM crops and mosquitoes as acceptable based on their positive experience with these mangoes; however grafting, in which the scions of one variety are fused to the root stock of another, does not constitute genetic modification. The term "genetically modified" is usually reserved for organisms modified by *in vitro *nucleic acid techniques that overcome natural physiological barriers [22].

Other organisms perceived as GMOs by participants were not genetically modified in the technical sense. In urban areas, a third of participants believed that their chickens were genetically modified; however these were more likely selectively bred chickens introduced from elsewhere. A few urban participants believed that their tomatoes, corn, rice and ground nuts had been genetically modified. In Tienfala, three villagers judged genetic engineering as acceptable because of their positive experience with artificial insemination in cows. It is important to appreciate these perceptions because it is within this context that people will judge the acceptability of GM mosquitoes.

### Conditions for a release of GM crops

Participants were pragmatic about the hypothetical release of pest-resistant crops such as insect-resistant GM corn. In rural areas, 18 out of 30 participants requested a trial be performed to confirm the crop's beneficial consequences and lack of negative consequences prior to a large-scale release. Of these participants, 16 wanted to conduct the trial themselves; while one wanted the trial to be conducted in a geographically similar environment. Some were very specific about the details of their proposed trial. As one participant described: "*I would choose a different space to culture the new crop, about one to two kilometres away from my farm. I would like this area to have the same area as my farm to provide a good comparison. Afterwards, I would collect the corn from the two farms and would see which produced the better yield*" (Excerpt from an interview with a 32-year-old man in Banambani). A trial period of one season was suggested and participants pledged to monitor the effects of GM corn on human health during this period.

A small number of rural participants preferred that the first trial be conducted in their village. As one participant stated: "*You have to start somewhere. From this, people will know whether it's good or bad... I would like you to conduct a trial in my village because I would like to be an example for another community*" (Excerpt from an interview with a 72-year-old man in Kaporo-na). The basic concept of the trial was that, before replacing their local variety with a modified variety of corn, villagers wanted to see for themselves that the modified variety produces a better yield with no negative side-effects for human health or the environment. A number of other conditions for a release of GM corn were given and are listed in Additional file 3.

### Conditions for a release of GM mosquitoes

Participants were open to the idea of a release of GM mosquitoes for malaria control subject to conditions - 62 said they would support a release that satisfied their conditions, 14 said they would not support a release under any circumstances, and four were unsure. These figures may be misleading since not all of the participants' conditions will be achievable; however it is noteworthy that only 14 out of 80 participants were unconditionally against a release. For comparison, in a recent survey of attitudes to biotechnology in Japan [18], 20% said that they would not support "a release of GM mosquitoes which do not transmit human disease."

The concept of a trial for GM crops was reapplied to a release of GM mosquitoes. One participant explicitly related the two together: "*I would have to see an example of modified mosquitoes reducing malaria in another village before I believe this claim of the foreign organization. In agriculture, you have to see something before you believe it*" (Excerpt from an interview with a 72-year-old man in Tienfala). In rural areas, one participant wanted to conduct the trial in their own village while seven wanted the trial to be held in another village, preferably in a geographically similar environment. Two participants requested bed nets to reduce contact between people and GM mosquitoes until the modified mosquitoes have been proven to work. Another requested that a hospital be built in their village to treat people who experience negative health effects.

Prior to a village trial, seven rural participants required that laboratory experiments be conducted and results provided to confirm that GM mosquitoes are able to reduce malaria prevalence. The GM strains were requested to be modified from local mosquitoes so they are adapted to the local environment. One participant mentioned that the intervention should be free for the local community, and that it should not cause villagers to spend any more money on insecticides or anti-malarial drugs. Urban participants were particularly conscious of human health effects, with four requesting evidence that GM mosquitoes will not transmit other diseases, including AIDS. Seven participants said that they wanted doctors and scientists to address these risks, with three requesting a dialogue between the community and scientists, including African scientists. Many were eager to understand how the technology works. Another participant required it to be possible to abort the project in the event of a serious mishap. Additional conditions, including those of health professionals, are listed in Table 3.

**Table 3:** Conditions for a release of GM mosquitoes.

	Conditions for a release of GM mosquitoes
Rural areas	Trial to confirm safety and efficacy (in own village or similar environment) Laboratory experiments to confirm safety and efficacy Access to experimental results Assurance from foreign organization, local medical staff, Malian government, United Nations Foreign organization works with Malian government, local medical staff Mosquito strains captured from local environment Bed nets provided, hospital built prior to a release Education campaign Approval by majority of community No cost to community

Urban areas	Evidence that GM mosquitoes will not cause human health concerns, transmit other diseases, transmit AIDS Trial to confirm safety and efficacy (in another location) Laboratory experiments to confirm safety and efficacy Ability to abort project Education campaign Dialogue between community and scientists Foreign scientists work with African scientists, United Nations Assurance from foreign organization, Malian government Approval by majority of community No cost to community Aid money given directly to scientists

Doctors & scientists	Evidence that GM mosquitoes will not cause human health concerns, environmental concerns, transmit other diseases Laboratory experiments to confirm safety and efficacy Communication of experimental results Education campaign Prevention of malaria parasite developing resistance to antimalarial gene No cost to community

Traditional healers	Evidence that GM mosquitoes will not cause human health concerns, have increased ability to transmit malaria Technology accessible to all communities Assurance from United Nations, foreign exporter, local experts GM mosquitoes developed and tested in Africa Foreign organization works with Malian government GM mosquitoes also developed for other diseases No cost to community

Necessary conditions for a release of GM mosquitoes cited by rural and urban populations, doctors, scientists and traditional healers.

### Balancing the risks of malaria and malaria control

Several responses to the questions on GM mosquitoes compared the risks of genetic engineering to the risks of malaria itself: "*If you can do it [engineer mosquitoes unable to carry malaria], it will be better, because malaria is more dangerous than any other consequences that this project could have*" (Excerpt from an interview with a 36-year-old woman in Sans Fil). The same sentiment was expressed towards malaria control strategies using insecticides: "*If we kill mosquitoes using chemicals, we might get headaches; but this is still better than getting malaria*" (Excerpt from an interview with a 28-year-old woman in Baco Djikoroni). Another participant said that rheumatism would be an acceptable side-effect because he considered malaria to be worse than rheumatism.

### Preference for killing or modifying mosquitoes

Many participants preferred that mosquitoes be killed rather than modified; however it is important to note that there was a range of preferences for killing rather than modifying mosquitoes. In rural areas, for example, there were seven participants who didn't believe that GM mosquitoes could reduce malaria prevalence at all and favored killing mosquitoes under all circumstances. Another five participants preferred that mosquitoes be killed but were open to a release of GM mosquitoes under certain conditions, for example: "*For me, a mosquito is a mosquito; but if an organization tells me that these mosquitoes will be good for my community in the fight against malaria, I will trust them*" (Excerpt from an interview with a 65-year-old woman in Kaporo-na). Another participant began by asking why the organization can't kill all mosquitoes and then conceded that he would accept a release "*because there is no way to kill all mosquitoes so the new mosquitoes are better if they don't transmit malaria*" (Excerpt from an interview with a 68-year-old man in Kaporo-na). A similar range of responses were given in urban areas.

### Concerns about GM mosquitoes

The main concern expressed by participants in all groups, but particularly amongst those from rural areas, was that the strategy of releasing GM mosquitoes will not work (Table 4). As one participant asked: "*After this work on modified mosquitoes, will there be a good solution for the malaria burden?*" (Excerpt from an interview with a 72-year-old man in Tienfala). A related concern expressed by three traditional healers and a local scientist was that GM mosquitoes will only partially replace the wild mosquito population. A doctor was also concerned that the malaria parasite will develop resistance to the anti-malarial gene.

**Table 4:** Concerns about GM mosquitoes.

General concerns about the GM mosquito project	Total*
Project will not work	
• GM mosquitoes will transmit malaria like wild mosquitoes	30
• Offspring of wild and GM mosquitoes will transmit malaria	
• A release will therefore lead to more malaria-transmitting mosquitoes	
• GM mosquitoes won't be adapted to conditions in Mali/Africa	
• Malaria parasite will develop resistance to the antimalarial gene	
• GM mosquitoes will only partially solve the malaria problem	

Human health concerns	
• GM mosquitoes will transmit other diseases	25
• GM mosquitoes will transmit AIDS	
• GM mosquitoes will be resistant to insecticides	
• GM mosquitoes will transmit a strain of malaria for which humans have no acquired immunity	
• Accidentally eating GM mosquitoes will cause illness	
• GM mosquitoes won't transmit diseases where they are developed; but will transmit diseases in Mali/Africa	

General bad consequences	
• GM mosquitoes won't cause problems where they are developed; but will cause problems in Mali/Africa	14
• GM mosquitoes will adapt and become dangerous	

Environmental concerns	6

Cost to the community	4

* Number of interviewees

A related concern voiced by several rural participants was that, if the strategy does not work, then a release could actually increase malaria prevalence by releasing even more malaria-transmitting mosquitoes. One rural participant was concerned that the offspring of wild and GM mosquitoes would transmit malaria. There was also a concern that GM mosquitoes will be developed and tested in Western countries and not adapted to local conditions in Mali or the rest of Africa. One of the traditional healers articulated this concern in the following statement: "*Even if they are not transmitting diseases like the mosquitoes in the United States and Europe, when they get here they can adapt to the local conditions and they can start transmitting diseases again*" (Excerpt from an interview with a 52-year-old man in Bandiagara).

The second main concern about GM mosquitoes was their effect on human health. Several participants expressed the concern that GM mosquitoes will transmit other diseases, as the following statement illustrates: "*Malaria is a disease that is transmitted via the blood; and another such disease that I am afraid of is AIDS*" (Excerpt from an interview with a 35-year-old man in Baco Djikoroni). Traditional healers expressed concerns about increased malaria transmission due to GM mosquitoes being more resistant to insecticides or transmitting strains of malaria that humans have not acquired immunity against. One rural participant expressed concern over the consequences of ingesting a GM mosquito: "*Even if the new mosquitoes don't transmit diseases by biting; what will happen if we eat them or drink them accidentally in water*" (Excerpt from an interview with a 28-year-old man in Kaporo-na). Less commonly expressed concerns were negative consequences for the environment, mentioned by six participants, and the cost of a transgenic release, mentioned by four participants.

### The decision-making process

A major problem facing democracy in rural communities is the lack of technical expertise required to make informed decisions. To address this, several participants suggested creating a science course on the subject of GM mosquitoes, funding education and discussion groups, and generally increasing living standards to empower people. Another participant suggested talking to the local media to increase awareness.

Another critical issue is the involvement of women in decision-making. As one of the wives interviewed observed: "*In our community, women are not involved in making decisions. This is the husband's role and he doesn't discuss decisions with his wife*" (Excerpt from an interview with a 34-year-old woman in Sans Fil). There are some indications that the Malian family structure is becoming more democratic: "*Together, we [myself and my husband] make a decision, and after that we call the rest of the family - the children - and inform them...*" (Excerpt from an interview with a 60-year-old woman in Sans Fil). However, it remains that village chiefs are always male, and there is at most one woman who can vote on village matters.

### Trust in local and foreign organizations

Among those interviewed, the most-trusted organizations on issues related to biotechnology were the Malian government, the United Nations and scientific organizations. The Malian government was most trusted in rural communities, with several participants citing government approval as the only necessary requirement for a release of GMOs. Scientific organizations were most trusted in urban communities and among doctors and scientists. United Nations organizations were fairly uniformly trusted among all groups (Additional file 4).

## Discussion

Public attitudes to disease control strategies using GM mosquitoes are particularly important given both the controversy surrounding GMOs and the potentially dangerous health consequences of modifying a disease vector. This study suggests that, while a release of GM mosquitoes was unacceptable to some of the people interviewed, most participants were open to a strategy that can be shown to be effective without significant negative consequences for human health and the environment. There was widespread desire to see evidence that GM mosquitoes can reduce malaria prevalence, preferably through the performance of a trial. Preference for a trial was most common in rural communities, where participants drew parallels to agriculture and demanded similar requirements of both GM crops and GM mosquitoes, although they were more sceptical of the latter. For several people, the concept of modifying a mosquito was unbelievable and a mosquito control programme was preferable; however, even these participants were often partial to a release of GM mosquitoes provided that the release would satisfy their requirements.

### Interpretation of survey results

In general, the public surveys were very well-received. One of the urban participants expressed his gratitude in the following statement: "*Normally, people go straight to the government for their projects, but you start with the community, so thank you*" (Excerpt from an interview with a 55-year old man in Baco Djikoroni). Indeed, it is essential to engage the community in the early stages of the research when the protocol is being development. However, one must be very careful when interpreting these results. The survey was far from a random sample. In rural areas, appropriate people to interview were suggested by the village chief. This led to a bias towards council members, other decision-makers and men. In the suburbs of Bamako, there was a bias towards heads of families and people who were home at the time of the interview. Furthermore, both villages and suburbs were selected by recommendation. Doctors, scientists and traditional healers were also selected by recommendation, and people in these professions are usually men. Hence, the collective responses may be considered a subset of the public opinion in Mali; but not necessarily a representative one. Nevertheless they do provide an idea of the range of perspectives across the country, which will be of use in the development of subsequent quantitative surveys.

In rural areas, interviews were conducted over several days, allowing early participants time to talk to those interviewed later. The chief had an opportunity to influence results, being both the first interviewee in a village and being involved in the selection of subsequent interviewees. Analysis of survey results does not reveal a pattern of participants repeating the chief's perspective; however this concern should be considered. Another concern is that participants generally preferred to answer questions in their living quarters surrounded by friends and relatives. This may have made them reluctant to give controversial responses that could have led to social stigma. Imperato [23] suggested this to be the reason why so many people identify as Muslim in public censuses, despite still having strong Animist beliefs.

A local Malian translator was always present to pose questions and translate responses; however several essential words, such as "gene", "genetic engineering" and "biotechnology", were difficult to explain in local dialects. It is therefore important to understand what participants understood of these terms and the context in which they gave their responses. Over half of the urban and rural participants cited God and/or sharing the same blood as the reason why human offspring tend to resemble their parents. Being primarily agricultural, they also demonstrated a strong practical knowledge of heredity through selective breeding. Several urban and rural participants were familiar with GMOs, terminator seed technology and the GM cotton controversy in Burkina Faso, while still believing in blood as the hereditary material of humans. No distinction was made between modern genetic engineering and agricultural and reproductive technologies such as grafting and artificial insemination. It was in this context that participants offered their opinions on GM mosquitoes.

Many participants perceived mosquitoes to be one of several causes of malaria, and some did not believe mosquitoes to be involved in malaria transmission at all. It is interesting to note that this did not seem to influence their support for a release of GM mosquitoes. Of eight participants who did not include mosquitoes as one of the main causes of malaria, four supported a release of GM mosquitoes, three were partial to a release, and only one was opposed to a release. The ratios are similar for people who cited mosquitoes as the sole main cause or one of several main causes of malaria.

A similar point has been made by Macer [24], based on his public attitude surveys to biotechnology in New Zealand and Japan [15,16,18], which found that the relationship between knowledge and acceptance is not linear. This survey concurs with those of Macer [15], in that Western-trained doctors and scientists are no more supportive of GM mosquitoes or biotechnology than the general population, despite their superior education in science, genetics and disease transmission.

Coercion is another issue that should be considered when interpreting public attitudes to medical interventions. This was apparent in the village of Banambani, where participants were supportive of the GM mosquito project; but were also particularly demanding of a hospital. This expectation stemmed from a recent history in which the neighbouring village cooperated in a parasitological study and a small medical centre was built in their village to serve research purposes, but also as a form of compensation. Consequently, some interviewees in Banambani seemed to accept the GM mosquito project based on the expectation that they will get a hospital in return. In another sense, it represents a trust that the community will gain from participation in research; however the perceived benefit is independent of the research goal and leaves the population vulnerable to exploitation.

The limited participation of women should also be considered. Women represented a third of the sample, appeared to be more submissive than men, and often sought the advice of others to answer simple questions. It was more difficult to interview women in rural and urban settings because they were washing clothes, cleaning, looking after babies, getting water, preparing food and it was harder for them to take time out to be interviewed. Although some of the women interviewed were formally educated, they tended to be less well-educated than men. Wives seemed uncomfortable talking on their husband's behalf given that their husband is the head of the family; however, explaining the premise that the interview team would like to hear the opinions of both women and men was helpful. Future survey teams may benefit from having female interviewers and translators to interview women.

Finally, on a more fundamental level, it must be questioned how much can be concluded from a public attitudes survey to a project that has not yet been carried out. Participants noted: "*If you don't know something, you can't say whether it's important or not*" (Excerpt from an interview with a 36-year-old man in Sabalibougou). Additionally, it is known that people react differently to situations they consider real or hypothetical [24]. True opinions may only be retrospective; however preliminary inquiries are necessary to provide useful information to those developing trial protocols.

## Conclusions

In summary, the results of this study suggest that, although there were some dissenters, the majority of participants surveyed in rural and urban Mali were pragmatic and open to a release of GM mosquitoes for malaria control. Prior to a release, they would like to see evidence for themselves that the technology is safe and efficient, preferably through the performance of a trial. An array of social and cultural issues associated with malaria, mosquitoes and genetic engineering became apparent. If these can be successfully addressed, then social acceptance among the populations surveyed seems promising.

## Competing interests

The authors declare that they have no competing interests.

## Authors' contributions

JMM, MBT and CET designed the study. JMM, MBT, MMT and CET performed the public attitude surveys. JMM, SF and CET analyzed the survey data. JMM wrote the manuscript. All authors read and approved the final version of the manuscript.

## Supplementary Material

Additional file 1 Demographic information of rural and urban populations, doctors, scientists and traditional healers. Click here for file

Additional file 2 Recruitment letters, research information sheets and questionnaires for rural and urban populations, doctors, scientists and traditional healers. Click here for file

Additional file 3 Necessary conditions for a release of GM corn cited by rural and urban populations, doctors, scientists and traditional healers. Click here for file

Additional file 4 Most trusted organizations for issues related to biotechnology among rural and urban populations, doctors, scientists and traditional healers. Click here for file
