# Supplementary material for: Perspectives of people in Mali toward genetically-modified mosquitoes for malaria control
Source: Malar J. 2010 May 14;9:128. doi: 10.1186/1475-2875-9-128 (PMC2881074; doi:10.1186/1475-2875-9-128)
Supplement: Additional file 1 — Demographic information of rural and urban populations, doctors, scientists and traditional healers. [file 1475-2875-9-128-S1.DOC]

**Additional file 1 - Table - Demographic information**

|  | | Rural areas | Urban areas | Doctors & scientists | Traditional healers | Total |
| --- | --- | --- | --- | --- | --- | --- |
| Age range (years) | | 20-88 | 22-66 | 28-52 | 27-62 | 20-88 |
| Median age (years) | | 46 | 35 | 46 | 42 | 44 |
| Gender | Male^*^ | 21 | 18 | 7 | 7 | 53 |
|  | Female^*^ | 9 | 12 | 3 | 3 | 27 |
| Education | No formal education^*^ | 12 | 13 | – | 5 | 30 |
|  | Arabic school^*^ | 2 | 2 | – | – | 4 |
|  | Public school^*^ | 11 | 4 | – | 3 | 18 |
|  | University^*^ | 2 | 9 | 3 | – | 14 |
|  | Medical degree/PhD^*^ | – | – | 7 | – | 7 |
| Religion | Islam^*^ | 28 | 27 | 6 | 8 | 69 |
|  | Christianity^*^ | 2 | 2 | 2 | 2 | 8 |
|  | Atheism^*^ | – | – | 1 | – | 1 |

^*^ Number of interviewees
