# Supplementary material for: Perspectives of people in Mali toward genetically-modified mosquitoes for malaria control
Source: Malar J. 2010 May 14;9:128. doi: 10.1186/1475-2875-9-128 (PMC2881074; doi:10.1186/1475-2875-9-128)
Supplement: Additional file 2 — Recruitment letters, research information sheets and questionnaires for rural and urban populations, doctors, scientists and traditional healers. [file 1475-2875-9-128-S2.PDF]

APPROVAL NOTICE

OFFICE FOR PROTECTION OF RESEARCH SUBJECTS
11000 Kinross Avenue, Suite 102
169407
www.oprs.ucla.edu

DATE: August 11, 2008

TO: Charles E. Taylor, PhD
Principal Investigator

FROM: Alison A. Moore, M.D., M.P.H.
Chair, South General Institutional Review Board

RE: UCLA IRB #G08-04-081-01
Approved by Expedited Review
(Approval Period from 08/11/2008 **through** 08/07/2009)
Genetically Modified Mosquitoes for Malaria Control: Attitudes toward Biotechnology in Mali, West Africa

Please be notified that the UCLA Institutional Review Board (UCLA IRB) has approved the above referenced research project involving human subjects in research. The UCLA's Federalwide Assurance (FWA) with the Department of Health and Human Services, Office for Human Research Protections is FWA00004642.

PLEASE COMPLY WITH THE FOLLOWING CODICIL(S) IMPOSED BY THE IRB:

1. **The UCLA IRB acknowledges receipt of the attached translated copies of the approved English version of the study documents. The UCLA IRB accepts the translated copies of the currently approved English versions based upon the Principal Investigator's assurance of the accuracy of the content. The attached copies have been stamped with the expiration date only and can be used to recruit and consent French speaking subjects when needed.**

2. **Upon the expected enrollment of Bambara and Dogon speaking subjects, Bambara and Dogon recruitment materials and Information sheet(s) must be received and acknowledged by the UCLA IRB (through issuance of a revised approval notice) prior to implementation**

APPROVAL NOTICE
IRB #G08-04-081-01

Approval Signature of the UCLA IRB Chair

PRINCIPLES TO BE FOLLOWED BY PRINCIPAL INVESTIGATORS:

As the Principal Investigator, you have ultimate responsibility for the conduct of the study, the ethical performance of the project, the protection of the rights and welfare of human subjects, and strict adherence to any stipulations imposed by the UCLA IRB. You must abide by the following principles when conducting your research:

1. Perform the project by qualified personnel according to the approved protocol.
2. Do not implement changes in the approved protocol or consent form without prior UCLA IRB approval (except in a life-threatening emergency, if necessary to safeguard the well-being of human subjects.)
3. If written consent is required, obtain the legally effective written informed consent from human subjects or their legally responsible representative using only the currently approved UCLA-IRB stamped consent form.
4. Promptly report all undesirable and unintended, although not necessarily unexpected adverse reactions or events, that are the result of therapy or other intervention, within ten working days of occurrence. All fatal or life-threatening events must be reported to the UCLA IRB in writing within 2 working days after discovery.
5. In clinical medical research, any physician(s) caring for your research subjects must be fully aware of the protocol in which the subject is participating.
6. No subjects may be identified, contacted, recruited, or enrolled until the contract with the sponsor is finalized by the University.
7. Ensure that all individuals who will interact with subjects and/or have access to identifiable research data have completed the UCLA Protection of Human Research Subjects Certification.
8. Ensure that all individuals who will access subjects' medical records have completed the UCLA HIPAA Research Training Certification.
9. If non-UCLA sites or personnel are involved, follow all study-specific requirements and consent processes approved by the UCLA IRB.

FUNDING SOURCE(S):

According to the information provided in your application, the funding source(s) for this research project may include the following: extramural.

PI of Contract/Grant: Charles Taylor
Funding Source: NIH
Contract/Grant No: 1 R56 AI072549 (20060752)

APPROVAL NOTICE
IRB #G08-04-081-01

Contract/Grant Title: Dynamics of Transposable Elements in Mosquito Vectors

Malaria Research and Training Center
Faculty of Medicine
University of Bamako
Bamako, Mali

June 16, 2008

Re: Survey on attitudes toward biotechnology in Mali, West Africa

Dear community member,

We are writing to request your participation in a research study on the perspectives of the people of Mali to biotechnology.

You were selected as a possible participant in this study because the chief and elders of the village thought you would provide an insight into the questions we are interested in.

The questions are designed to gain an understanding of the views of Malians towards nature and biotechnology. Some of the questions also investigate the process by which decisions are made on these issues. The survey will take approximately half an hour to 45 minutes to complete, and will require only one session.

One potential application of the research will be to understand the acceptability of the use of genetically modified mosquitoes to control malaria in Mali. Your responses will help to inform an understanding of this.

If you are interested in taking the survey, we encourage you to contact one of the principal investigators directly. Our contact details are: Mahamadou Toure (phone: 223-677-8912, email: mah.toure@gmail.com, address: MRTC, Faculty of Medicine, University of Bamako, Bamako, Mali), and John Marshall (phone: 223-411-5334, email: john.macky.marshall@gmail.com, address: MRTC, Faculty of Medicine, University of Bamako, Bamako, Mali).

The survey will be conducted at a location of your choice. Questions will be asked either in spoken form in English, French, Bambara or Dogon; or in written form in English or French.

Thank you very much for taking the time to review this letter, and we will look forward to hearing from you.

Sincerely,

Mahamadou Toure, John Marshall and Samba Diop

APPROVED
AUG 11 2008
UCLA SOUTH GENERAL
INSTITUTIONAL REVIEW BOARD

Malaria Research and Training Center
Faculty of Medicine
University of Bamako
Bamako, Mali

June 16, 2008

Re: Survey on attitudes toward biotechnology in Mali, West Africa

Dear community member,

We are writing to request your participation in a research study on the perspectives of the people of Mali to biotechnology.

You were selected as a possible participant in this study because one of the representatives of a prominent institution in Bamako thought you would provide an insight into the questions we are interested in.

The questions are designed to gain an understanding of the views of Malians towards nature and biotechnology. Some of the questions also investigate the process by which decisions are made on these issues. The survey will take approximately half an hour to 45 minutes to complete, and will require only one session.

One potential application of the research will be to understand the acceptability of the use of genetically modified mosquitoes to control malaria in Mali. Your responses will help to inform an understanding of this.

If you are interested in taking the survey, we encourage you to contact one of the principal investigators directly. Our contact details are: Mahamadou Toure (phone: 223-677-8912, email: mah.toure@gmail.com, address: MRTC, Faculty of Medicine, University of Bamako, Bamako, Mali), and John Marshall (phone: 223-411-5334, email: john.macky.marshall@gmail.com, address: MRTC, Faculty of Medicine, University of Bamako, Bamako, Mali).

The survey will be conducted at a location of your choice. Questions will be asked either in spoken form in English, French, Bambara or Dogon; or in written form in English or French.

Thank you very much for taking the time to review this letter, and we will look forward to hearing from you.

Sincerely,

Mahamadou Toure, John Marshall and Samba Diop

APPROVED
AUG 1 1 2008
UCLA SOUTH GENERAL
INSTITUTIONAL REVIEW BOARD

Malaria Research and Training Center
Faculty of Medicine
University of Bamako
Bamako, Mali

June 16, 2008

Re: Survey on attitudes toward biotechnology in Mali, West Africa

Dear medical or research professional,

We are writing to request your participation in a research study on the perspectives of the people of Mali to biotechnology.

You were selected as a possible participant in this study because one of the head scientists at the Malaria Research and Training Center (MRTC) in Bamako thought you would provide an insight into the questions we are interested in.

The questions are designed to gain an understanding of the views of Malians towards nature and biotechnology. Some of the questions also investigate the process by which decisions are made on these issues. The survey will take approximately half an hour to 45 minutes to complete, and will require only one session.

One potential application of the research will be to understand the acceptability of the use of genetically modified mosquitoes to control malaria in Mali. Your responses will help to inform an understanding of this.

If you are interested in taking the survey, we encourage you to contact one of the principal investigators directly. Our contact details are: Mahamadou Toure (phone: 223-677-8912, email: mah.toure@gmail.com, address: MRTC, Faculty of Medicine, University of Bamako, Bamako, Mali), and John Marshall (phone: 223-411-5334, email: john.macky.marshall@gmail.com, address: MRTC, Faculty of Medicine, University of Bamako, Bamako, Mali).

The survey will be conducted at a location of your choice. Questions will be asked either in spoken form in English, French, Bambara or Dogon; or in written form in English or French.

Thank you very much for taking the time to review this letter, and we will look forward to hearing from you.

Sincerely,

Mahamadou Toure, John Marshall and Samba Diop

APPROVED
AUG 11 2008
UCLA SOUTH GENERAL
INSTITUTIONAL REVIEW BOARD

University of California, Los Angeles

RESEARCH INFORMATION SHEET

Genetically modified mosquitoes for malaria control:
Attitudes toward biotechnology in Mali, West Africa

You are asked to participate in a research study conducted by Mahamadou Toure, M.D., M.S., John Marshall, Ph.D., and Charles Taylor, Ph.D., from the Department of Ecology and Evolutionary Biology at the University of California, Los Angeles. You were selected as a possible participant in this study because the chief and elders of the village thought you would provide an insight into the questions we are interested in. Your participation in this research study is voluntary.

PURPOSE OF THE STUDY

To gain an insight into the attitudes of the people of Mali, West Africa toward biotechnology. To understand the concepts and values which inform these attitudes.

PROCEDURES

If you volunteer to participate in this study, we would ask you to do the following:

Answer the questions in the survey that follows. You may read the questions in English or French. You may also have the questions read to you in English, French, Bambara or Dogon. You may write the responses in English or French. You may also answer the questions by speaking in English, French, Bambara or Dogon. If you answer by speaking, your responses will be recorded using a dictaphone.

It is important to be honest in your responses to these questions. The information you provide may be referred to by decision-makers who are interested in your opinions. The survey will take between half an hour and 45 minutes, will require only one session and will be conducted at a location of your choice. If you answer in writing, you may complete the survey on your own and return it to the following address: Malaria Research and Training Center, Faculty of Medicine, University of Bamako, Bamako, Mali.

You are encouraged to ask questions if there is something in the survey that you don't understand. If there are any questions that you do not want to answer, you do not need to answer them.

POTENTIAL RISKS AND DISCOMFORTS

There are no direct physical or psychological risks to your participation in this survey. Despite this, it is possible that your colleagues or community members may act differently towards you if they discover that you have opinions that they disagree with. We will therefore ensure that your responses to the questions posed in this survey remain confidential.

APPROVED
AUG 11 2008
UCLA SOUTH GENERAL
INSTITUTIONAL REVIEW BOARD

POTENTIAL BENEFITS TO SUBJECTS AND/OR TO SOCIETY

You will not directly benefit from your participation in the research.

The results of the research may contribute to an understanding of the attitudes of the people of Mali, West Africa toward biotechnology. Biotechnology is the use of tools and methods based on biology. Examples are new medicines and vaccines. One potential application of biotechnology in Mali is the use of genetically modified mosquitoes to control malaria. Your responses will help to inform an understanding of the acceptability of this technology.

PAYMENT FOR PARTICIPATION

You will receive no payment for your participation.

CONFIDENTIALITY

The survey is anonymous. No identifying information will be recorded in association with your answers. The survey records will only be available to the investigators. Once the study has been completed, these records will be stored in locked cabinets at the MRTC in Bamako, with a digital copy on the principal investigator's computer at UCLA. This will be encrypted and password-protected.

PARTICIPATION AND WITHDRAWAL

You can choose whether to be in this study or not. If you volunteer to be in this study, you may withdraw at any time without consequences of any kind.

IDENTIFICATION OF INVESTIGATORS

If you have any questions or concerns about the research, please feel free to contact: Mahamadou Toure (phone: 223-677-8912, email: mah.toure@gmail.com, address: MRTC, Faculty of Medicine, University of Bamako, Bamako, Mali), John Marshall (phone: 223-411-5334, email: john.macky.marshall@gmail.com, address: MRTC, Faculty of Medicine, University of Bamako, Bamako, Mali), Prof Charles Taylor (phone: +1-310-206-3987, address: email: taylor@biology.ucla.edu, address: Department of Ecology and Evolutionary Biology, University of California, Los Angeles, CA 90095, USA).

RIGHTS OF RESEARCH SUBJECTS

You may withdraw your consent at any time and discontinue participation without penalty. You are not waiving any legal rights because of your participation in this research study. If you have questions regarding your rights as a research subject, contact the Institutional Review Board, Faculty of Medicine, University of Bamako, Pointe G, Bamako, Mali.

APPROVED
AUG 11 2008
UCLA SOUTH GENERAL
INSTITUTIONAL REVIEW BOARD

CONFIRMATION OF UNDERSTANDING

If this is a verbal rather than written agreement, please explain to us what we are agreeing to, in order to confirm that there are no misunderstandings.

"I have accurately explained the consent form completely at a level appropriate for the respondent to understand. The respondent has voluntarily agreed to participate in the study."

Signature of field-worker : Date

______________________________ : ___________

"I verify that the translation into the following language, ____________________ , is accurate."

Signature of translator : Date

______________________________ : ___________

UCLA IRB #G08-04-081-01
Expiration Date: August 7, 2009

APPROVED
AUG 11 2008
UCLA SOUTH GENERAL
INSTITUTIONAL REVIEW BOARD

University of California, Los Angeles

RESEARCH INFORMATION SHEET

Genetically modified mosquitoes for malaria control:
Attitudes toward biotechnology in Mali, West Africa

You are asked to participate in a research study conducted by Mahamadou Toure, M.D., M.S., John Marshall, Ph.D., and Charles Taylor, Ph.D., from the Department of Ecology and Evolutionary Biology at the University of California, Los Angeles. You were selected as a possible participant in this study because one of the head scientists at the Malaria Research and Training Center (MRTC) in Bamako thought you would provide an insight into the questions we are interested in. Your participation in this research study is voluntary.

PURPOSE OF THE STUDY

To gain an insight into the attitudes of the people of Mali, West Africa toward biotechnology. To understand the concepts and values which inform these attitudes.

PROCEDURES

If you volunteer to participate in this study, we would ask you to do the following:

Answer the questions in the survey that follows. You may read the questions in English or French. You may also have the questions read to you in English, French, Bambara or Dogon. You may write the responses in English or French. You may also answer the questions by speaking in English, French, Bambara or Dogon. If you answer by speaking, your responses will be recorded using a dictaphone.

It is important to be honest in your responses to these questions. The information you provide may be referred to by decision-makers who are interested in your opinions. The survey will take between half an hour and 45 minutes, will require only one session and will be conducted at a location of your choice. If you answer in writing, you may complete the survey on your own and return it to the following address: Malaria Research and Training Center, Faculty of Medicine, University of Bamako, Bamako, Mali.

You are encouraged to ask questions if there is something in the survey that you don't understand. If there are any questions that you do not want to answer, you do not need to answer them.

POTENTIAL RISKS AND DISCOMFORTS

There are no direct physical or psychological risks to your participation in this survey. Despite this, it is possible that your colleagues or community members may act differently towards you if they discover that you have opinions that they disagree with. We will therefore ensure that your responses to the questions posed in this survey remain confidential.

APPROVED
AUG 11 2008
UCLA SOUTH GENERAL
INSTITUTIONAL REVIEW BOARD

POTENTIAL BENEFITS TO SUBJECTS AND/OR TO SOCIETY

You will not directly benefit from your participation in the research.

The results of the research may contribute to an understanding of the attitudes of the people of Mali, West Africa toward biotechnology. Biotechnology is the use of tools and methods based on biology. Examples are new medicines and vaccines. One potential application of biotechnology in Mali is the use of genetically modified mosquitoes to control malaria. Your responses will help to inform an understanding of the acceptability of this technology.

PAYMENT FOR PARTICIPATION

You will receive no payment for your participation.

CONFIDENTIALITY

The survey is anonymous. No identifying information will be recorded in association with your answers. The survey records will only be available to the investigators. Once the study has been completed, these records will be stored in locked cabinets at the MRTC in Bamako, with a digital copy on the principal investigator's computer at UCLA. This will be encrypted and password-protected.

PARTICIPATION AND WITHDRAWAL

You can choose whether to be in this study or not. If you volunteer to be in this study, you may withdraw at any time without consequences of any kind.

IDENTIFICATION OF INVESTIGATORS

If you have any questions or concerns about the research, please feel free to contact: Mahamadou Toure (phone: 223-677-8912, email: mah.toure@gmail.com, address: MRTC, Faculty of Medicine, University of Bamako, Bamako, Mali), John Marshall (phone: 223-411-5334, email: john.macky.marshall@gmail.com, address: MRTC, Faculty of Medicine, University of Bamako, Bamako, Mali), Prof Charles Taylor (phone: +1-310-206-3987, address: email: taylor@biology.ucla.edu, address: Department of Ecology and Evolutionary Biology, University of California, Los Angeles, CA 90095, USA).

RIGHTS OF RESEARCH SUBJECTS

You may withdraw your consent at any time and discontinue participation without penalty. You are not waiving any legal rights because of your participation in this research study. If you have questions regarding your rights as a research subject, contact the Institutional Review Board, Faculty of Medicine, University of Bamako, Pointe G, Bamako, Mali.

APPROVED
AUG 1 1 2008
UCLA SOUTH GENERAL
INSTITUTIONAL REVIEW BOARD

CONFIRMATION OF UNDERSTANDING

If this is a verbal rather than written agreement, please explain to us what we are agreeing to, in order to confirm that there are no misunderstandings.

"I have accurately explained the consent form completely at a level appropriate for the respondent to understand. The respondent has voluntarily agreed to participate in the study."

Signature of field-worker : Date

_____________________________ : ___________

"I verify that the translation into the following language, ____________________, is accurate."

Signature of translator : Date

_____________________________ : ___________

UCLA IRB #G08-04-081-01
Expiration Date: August 7, 2009

APPROVED
AUG 1 1 2008
UCLA SOUTH GENERAL
INSTITUTIONAL REVIEW BOARD

University of California, Los Angeles

RESEARCH INFORMATION SHEET

Genetically modified mosquitoes for malaria control:
Attitudes toward biotechnology in Mali, West Africa

You are asked to participate in a research study conducted by Mahamadou Toure, M.D., M.S., John Marshall, Ph.D., and Charles Taylor, Ph.D., from the Department of Ecology and Evolutionary Biology at the University of California, Los Angeles. You were selected as a possible participant in this study because one of the representatives of a prominent institution in Bamako thought you would provide an insight into the questions we are interested in. Your participation in this research study is voluntary.

PURPOSE OF THE STUDY

To gain an insight into the attitudes of the people of Mali, West Africa toward biotechnology. To understand the concepts and values which inform these attitudes.

PROCEDURES

If you volunteer to participate in this study, we would ask you to do the following:

Answer the questions in the survey that follows. You may read the questions in English or French. You may also have the questions read to you in English, French, Bambara or Dogon. You may write the responses in English or French. You may also answer the questions by speaking in English, French, Bambara or Dogon. If you answer by speaking, your responses will be recorded using a dictaphone.

It is important to be honest in your responses to these questions. The information you provide may be referred to by decision-makers who are interested in your opinions. The survey will take between half an hour and 45 minutes, will require only one session and will be conducted at a location of your choice. If you answer in writing, you may complete the survey on your own and return it to the following address: Malaria Research and Training Center, Faculty of Medicine, University of Bamako, Bamako, Mali.

You are encouraged to ask questions if there is something in the survey that you don't understand. If there are any questions that you do not want to answer, you do not need to answer them.

POTENTIAL RISKS AND DISCOMFORTS

There are no direct physical or psychological risks to your participation in this survey. Despite this, it is possible that your colleagues or community members may act differently towards you if they discover that you have opinions that they disagree with. We will therefore ensure that your responses to the questions posed in this survey remain confidential.

APPROVED
AUG 11 2008
UCLA SOUTH GENERAL
INSTITUTIONAL REVIEW BOARD

POTENTIAL BENEFITS TO SUBJECTS AND/OR TO SOCIETY

You will not directly benefit from your participation in the research.

The results of the research may contribute to an understanding of the attitudes of the people of Mali, West Africa toward biotechnology. Biotechnology is the use of tools and methods based on biology. Examples are new medicines and vaccines. One potential application of biotechnology in Mali is the use of genetically modified mosquitoes to control malaria. Your responses will help to inform an understanding of the acceptability of this technology.

PAYMENT FOR PARTICIPATION

You will receive no payment for your participation.

CONFIDENTIALITY

The survey is anonymous. No identifying information will be recorded in association with your answers. The survey records will only be available to the investigators. Once the study has been completed, these records will be stored in locked cabinets at the MRTC in Bamako, with a digital copy on the principal investigator's computer at UCLA. This will be encrypted and password-protected.

PARTICIPATION AND WITHDRAWAL

You can choose whether to be in this study or not. If you volunteer to be in this study, you may withdraw at any time without consequences of any kind.

IDENTIFICATION OF INVESTIGATORS

If you have any questions or concerns about the research, please feel free to contact: Mahamadou Toure (phone: 223-677-8912, email: mah.toure@gmail.com, address: MRTC, Faculty of Medicine, University of Bamako, Bamako, Mali), John Marshall (phone: 223-411-5334, email: john.macky.marshall@gmail.com, address: MRTC, Faculty of Medicine, University of Bamako, Bamako, Mali), Prof Charles Taylor (phone: +1-310-206-3987, address: email: taylor@biology.ucla.edu, address: Department of Ecology and Evolutionary Biology, University of California, Los Angeles, CA 90095, USA).

RIGHTS OF RESEARCH SUBJECTS

You may withdraw your consent at any time and discontinue participation without penalty. You are not waiving any legal rights because of your participation in this research study. If you have questions regarding your rights as a research subject, contact the Institutional Review Board, Faculty of Medicine, University of Bamako, Pointe G, Bamako, Mali.

APPROVED
AUG 11 2008
UCLA SOUTH GENERAL
INSTITUTIONAL REVIEW BOARD

CONFIRMATION OF UNDERSTANDING

If this is a verbal rather than written agreement, please explain to us what we are agreeing to, in order to confirm that there are no misunderstandings.

"I have accurately explained the consent form completely at a level appropriate for the respondent to understand. The respondent has voluntarily agreed to participate in the study."

Signature of field-worker : Date

_____________________________ : __________

"I verify that the translation into the following language, ____________________ , is accurate."

Signature of translator : Date

_____________________________ : __________

UCLA IRB #G08-04-081-01
Expiration Date: August 7, 2009

APPROVED
AUG 11 2008
UCLA SOUTH GENERAL
INSTITUTIONAL REVIEW BOARD

Malaria Research and Training Center
Faculte de Medecine, Pharmacie et d'Odontostomatologie (FMPOS)
Universite de Bamako
Bamako, Mali

Le 16 Juin 2008

Re: Enquete sur les attitudes par rapport a la biotechnologie au Mali

Cher membre de la communauté,

Par la presente lettre nous venons vous demander de participer a une etude que nous menons sur les perceptions des populations du Mali sur la biotechnologie.

Vous aviez ete selectionne comme possible participant parceque les reponsables d'une imminente institution pense que vous pouvez apporter des reponses adequates aux questions qui nous ineteressent.

Les questions ont ete concues pour obtenir a la fin une analyse de la perception des Maliens par rapport a la nature et a la biotechnologie. Quelques questions ont trait aussi au processus par lequel sont prises les decision dans des situations similaires. L'enquete va prendre a peu pres une demie heure ou quarante cinq minutes et ne consiste qu'une seule cession.

L'application potentielle de cette recherche serait de comprendre l'acceptabilite de l'utilisation des moustiques genetiquement modifies pour lutter contre le paludisme au Mali. Vos reponses aideront pour une analyse de la situation

Si vous desirez prendre part a cette enquete, nous vous demandons de prendre contact avec un des principaux investigateurs directement. Il s'agit de Dr Mahamoudou Toure Tel: 223-677-8912, email: mah.toure@gmail.com, address: MRTC, Faculty of Medicine, University of Bamako, Bamako, Mali), et Dr John Marshall (Tel: 223-411-5334, email: john.macky.marshall@gmail.com, address: MRTC, Faculty of Medicine, University of Bamako, Bamako, Mali).

L'enquete se deroulera a l'endroit de cotre choix. Les questions sont posees en Anglais ou Francais. Elles peuvent aussi etre repondue par ecrit dans le deux langues sus citees.

Merci pour votre temps, et nous comptons sur une reaction de votre part dans les jours a venir.

Sincerement

Mahamadou Toure, John Marshall et Samba Diop

Malaria Research and Training Center
Faculte de Medecine, Pharmacie et d'Odontostomatologie (FMPOS)
Universite de Bamako
Bamako, Mali

Le 16 Juin 2008

Re: Enquete sur les attitudes par rapport a la biotechnologie au Mali

Cher membre de la communauté,

Par la presente lettre nous venons vous demander de participer a une etude que nous menons sur les perceptions des populations du Mali sur la biotechnologie.

Vous aviez ete selectionne comme possible participant parceque le chef de village et ses conseillers pense que vous pouvez apporter des reponses adequates aux questions qui nous ineteressent.

Les questions ont ete concues pour obtenir a la fin une analyse de la perception des Maliens par rapport a la nature et a la biotechnologie. Quelques questions ont trait aussi au processus par lequel sont prises les decision dans des situations similaires. L'enquete va prendre a peu pres une demie heure ou quarante cinq minutes et ne consiste qu'une seule cession.

L'application potentielle de cette recherche serait de comprendre l'acceptabilite de l'utilisation des moustiques genetiquement modifies pour lutter contre le paludisme au Mali. Vos reponses aideront pour une analyse de la situation

Si vous desirez prendre part a cette enquete, nous vous demandons de prendre contact avec un des principaux investigateurs directement. Il s'agit de Dr Mahamoudou Toure Tel: 223-677-8912, email: mah.toure@gmail.com, address: MRTC, Faculty of Medicine, University of Bamako, Bamako, Mali), et Dr John Marshall (Tel: 223-411-5334, email: john.macky.marshall@gmail.com, address: MRTC, Faculty of Medicine, University of Bamako, Bamako, Mali).

L'enquete se deroulera a l'endroit de cotre choix. Les questions sont posees en Anglais ou Francais. Elles peuvent aussi etre repondue par ecrit dans le deux langues sus citees.

Merci pour votre temps, et nous comptons sur une reaction de votre part dans les jours a venir.

Sincerement

Mahamadou Toure, John Marshall et Samba Diop

Malaria Research and Training Center
Faculte de Medecine, Pharmacie et d'Odontostomatologie (FMPOS)
Universite de Bamako
Bamako, Mali

Le 16 Juin 2008

Re: Enquete sur les attitudes par rapport a la biotechnologie au Mali

Cher Medecin/Chercheurs,

Par la presente lettre nous venons vous demander de participer a une etude que nous menons sur les perceptions des populations du Mali sur la biotechnologie.

Vous aviez ete selectionne comme possible participant parceque le Directeur scientifique du MRTC/FMPOS de Bamako pense que vous pouvez apporter des reponses adequates aux questions qui nous ineteressent.

Les questions ont ete concues pour obtenir a la fin une analyse de la perception des Maliens par rapport a la nature et a la biotechnologie. Quelques questions ont trait aussi au processus par lequel sont prises les decision dans des situations similaires. L'enquete va prendre a peu pres une demie heure ou quarante cinq minutes et ne consiste qu'une seule cession.

L'application potentielle de cette recherche serait de comprendre l'acceptabilite de l'utilisation des moustiques genetiquement modifies pour lutter contre le paludisme au Mali. Vos reponses aideront pour une analyse de la situation

Si vous desirez prendre part a cette enquete, nous vous demandons de prendre contact avec un des principaux investigateurs directement. Il s'agit de Dr Mahamoudou Toure Tel: 223-677-8912, email: mah.toure@gmail.com, address: MRTC, Faculty of Medicine, University of Bamako, Bamako, Mali), et Dr John Marshall (Tel: 223-411-5334, email: john.macky.marshall@gmail.com, address: MRTC, Faculty of Medicine, University of Bamako, Bamako, Mali).

L'enquete se deroulera a l'endroit de cotre choix. Les questions sont posees en Anglais ou Francais. Elles peuvent aussi etre repondue par ecrit dans le deux langues sus citees.

Merci pour votre temps, et nous comptons sur une reaction de votre part dans les jours a venir.

Sincerement

Mahamadou Toure, John Marshall et Samba Diop

University of California, Los Angeles

FICHE D'INFORMATION SUR LA RECHERCHE

Moustiques Genetiquement modifies pour la lutte contre le Paludisme:
Attitudes par rapport a la biotechnologie au Mali, Afrique de l'Ouest

Nous venons vous demander de bien vouloir participer a une etude menee par Dr Mahmoudou Toure, Dr John Marshall et Dr Charles Taylor du departement de biologie et d'ecologie de l'Universite de California a Los Angeles. Vous aviez ete selectionne pour participer a cette etude parceque les reponsables d'une imminente institution ont cru necessaires d'obtenir vos idees et conceptions sur les questions specifiques qui nous interessent. Votre participation est totalement volontaire.

OBJECTIVE DE L'ETUDE:

Obtenir un apercu general de l'attitude du people malien par rapport a la biotechnologie. Il s'agit de comprendre les concepts et les valeurs qui accompagnent cette attitude.

PROCEDURES

Si vous accepter de participer a notre etude, nous allons vous pose quelques questions:

Nous vous prions de repondre aux questions qui suivent. Vous pouvez lire les questions en anglais ou en francais et il en est de meme pour les reponses (cela est a votre choix). Mais si vous preferez repondre oralement, nous allons enregistrer vos reponses sur un Dictaphone.

Il est tres important d'etre honnete dans vos reponses a chaque question. Les informations recueillies peuvent etre soumises aux decideurs qui sont interesses par vos opinions. L'enquete peut prendre 30 a 45 mn et se fait seulement une fois et a votre lieu de preference. Mais si vous vous voulez ecrire vos reponses, vous avez la possibilite de remplir le questionnaire quand et ou vous voulez et nous les envoyer au MRTC/FMPOS.

Si vous etes confus par rapport au questionnaire, nous vous encourageons a nous les poser a tout moment. Aussi si vous ne voulez pas repondre a une question specifique, vous pouvez tout simplement continuer avec la suivante et laissez la place vide.

RISQUES POTENTIELS OU NON-CONFORTABLES:

Il n'y a aucun risqué direct ou psychologique par rapport a votre participation a l'etude. Au dela de tout ca, il est possible que vos collegues ou members de votre communite agissent differement s'ils decouvrent que vos opinions different des leurs. Nous vous rassurons qu'il y aura une confidentiallite totale en ce qui concerne vos reponses pendant et après l'etude.

BENEFICES:

Vous n'allez pas directement beneficie de votre participation a cette recherche.

Les resultants de cette etude peuvent contribuer a la comprehension des attitudes des populations du Mali par rapport a la biotechnologie. La biotechnologie est l'utilisation d'outils et de méthodes basées sur la biologie, e.g. nouveaux médicaments et vaccins. Un benefice potentiel serait l'utilisation des moustiques genetiquement modifies au Mali pour la lutte contre le paludisme. Vos reponses peuvent aider a eclairer l'acceptabilite de cette technologe.

REMUNERATION

Aucun participant ne serait payer pour sa participation a cette etude.

CONFIDENTIALITE

Cette enquete est totalement anonyme. Aucun identifiant ne serait associe aux informations obtenues. Les enregistrements seront seront accessibles aux investigateurs seuls. Une fois l'ettude terminee, ces enregistrements seront mis dans une armoire fermee a clef au MRTC, avec une copie numerique dans le bureau du principal ionvestigateur a UCLA. Elles seront encryptees et protégées par un mot de passe secret.

PARTICIPATION AND DESISTEMENT

Vous pouvez choisir de participer ou pas a cette etude. Si vous acceptiez volontier d'y participer, vous pouvez vous retirer a tout moment. Ce retrait est sans consequence pour vous.

IDENTIFICATION DES INVESTIGATEURS

Si vous avez une question concernant cette etude, nous vous prions de contacter le Dr Mahamoudou Toure (Tel: 223 6778912 ou email: mah.toure@gmail.com, addresse: MRTC, Faculty of Medicine, University of Bamako, Bamako, Mali), John Marshall (phone: 223-411-5334, email: john.macky.marshall@gmail.com, address: MRTC, Faculty of Medicine, University of Bamako, Bamako, Mali), Prof Charles Taylor (phone: +1-310-206-3987, address: email: taylor@biology.ucla.edu, address: Department of Ecology and Evolutionary Biology, University of California, Los Angeles, CA 90095, USA).

DROITS:

Vous pouvez vous retirer à tout moment et decider de re-participer sans aucune consequence. Vous ne renoncer a aucun droit legal a cause de votre participation a cette etude. Si vous avez des questions regardant votre droit en tant que participant a une etude de recherché vous pouvez contacter: IRB bureau de la FMPOS/Universite de Bamako au 222 7752 Universite de Bamako (MALI). BP: 1805 Tel 223 675 44 24

CONFIRMATION DE LA COMPREHENSION:

S'il s'agit d'une verbale plutôt que par accord écrit, s'il vous plaît nous expliquer ce que nous sommes d'accord, afin de confirmer qu'il n'y ait pas de malentendus.

SIGNATURE DE L'INVESTIGATEUR OU L'AUTORISE:

J'ai clairement expliqué la forme de consentement éclaire a un niveau approprie pour que le répondant puisse comprendre. Il (le répondant) a volontairement accepté de participe a cette étude.

Signature de l'enquêteur

______________________________ Date______________

J'ai vérifié que la traduction dans les langues suivantes ____________________ était claire.

Signature du traducteur

______________________________ Date______________

University of California, Los Angeles

FICHE D'INFORMATION SUR LA RECHERCHE

Moustiques Genetiquement modifies pour la lutte contre le Paludisme:
Attitudes par rapport a la biotechnologie au Mali, Afrique de l'Ouest

Nous venons vous demander de bien vouloir participer a une etude menee par Dr Mahmoudou Toure, Dr John Marshall et Dr Charles Taylor du departement de biologie et d'ecologie de l'Universite de California a Los Angeles. Vous aviez ete selectionne pour participer a cette etude parceque le chef de village et ses conseillers ont cru necessaires d'obtenir vos idees et conceptions sur les questions specifiques qui nous interessent. Votre participation est totalement volontaire.

OBJECTIVE DE L'ETUDE:

Obtenir un apercu general de l'attitude du people malien par rapport a la biotechnologie. Il s'agit de comprendre les concepts et les valeurs qui accompagnent cette attitude.

PROCEDURES

Si vous accepter de participer a notre etude, nous allons vous pose quelques questions:

Nous vous prions de repondre aux questions qui suivent. Vous pouvez lire les questions en anglais ou en francais et il en est de meme pour les reponses (cela est a votre choix). Mais si vous preferez repondre oralement, nous allons enregistrer vos reponses sur un Dictaphone.

Il est tres important d'etre honnete dans vos reponses a chaque question. Les informations recueillies peuvent etre soumises aux decideurs qui sont interesses par vos opinions. L'enquete peut prendre 30 a 45 mn et se fait seulement une fois et a votre lieu de preference. Mais si vous vous voulez ecrire vos reponses, vous avez la possibilite de remplir le questionnaire quand et ou vous voulez et nous les envoyer au MRTC/FMPOS.

Si vous etes confus par rapport au questionnaire, nous vous encourageons a nous les poser a tout moment. Aussi si vous ne voulez pas repondre a une question specifique, vous pouvez tout simplement continuer avec la suivante et laissez la place vide.

RISQUES POTENTIELS OU NON-CONFORTABLES:

Il n'y a aucun risqué direct ou psychologique par rapport a votre participation a l'etude. Au dela de tout ca, il est possible que vos collegues ou members de votre communite agissent differement s'ils decouvrent que vos opinions different des leurs. Nous vous rassurons qu'il y aura une confidentiallite totale en ce qui concerne vos reponses pendant et après l'etude.

BENEFICES:

Vous n'allez pas directement beneficie de votre participation a cette recherche.

Les resultants de cette etude peuvent contribuer a la comprehension des attitudes des populations du Mali par rapport a la biotechnologie. La biotechnologie est l'utilisation d'outils et de méthodes basées sür la biologie, e.g. nouveaux médicaments et vaccins. Un benefice potentiel serait l'utilisation des moustiques genetiquement modifies au Mali pour la lutte contre le paludisme. Vos reponses peuvent aider a eclairer l'acceptabilite de cette technologe.

REMUNERATION

Aucun participant ne serait payer pour sa participation a cette etude.

CONFIDENTIALITE

Cette enquete est totalement anonyme. Aucun identifiant ne serait associe aux informations obtenues. Les enregistrements seront seront accessibles aux investigateurs seuls. Une fois l'ettude terminee, ces enregistrements seront mis dans une armoire fermee a clef au MRTC, avec une copie numerique dans le bureau du principal ionvestigateur a UCLA. Elles seront encryptees et protégées par un mot de passe secret.

PARTICIPATION AND DESISTEMENT

Vous pouvez choisir de participer ou pas a cette etude. Si vous acceptiez volontier d'y participer, vous pouvez vous retirer a tout moment. Ce retrait est sans consequence pour vous.

IDENTIFICATION DES INVESTIGATEURS

Si vous avez une question concernant cette etude, nous vous prions de contacter le Dr Mahamoudou Toure (Tel: 223 6778912 ou email: mah.toure@gmail.com, addresse: MRTC, Faculty of Medicine, University of Bamako, Bamako, Mali), John Marshall (phone: 223-411-5334, email: john.macky.marshall@gmail.com, address: MRTC, Faculty of Medicine, University of Bamako, Bamako, Mali), Prof Charles Taylor (phone: +1-310-206-3987, address: email: taylor@biology.ucla.edu, address: Department of Ecology and Evolutionary Biology, University of California, Los Angeles, CA 90095, USA).

DROITS:

Vous pouvez vous retirer à tout moment et decider de re-participer sans aucune consequence. Vous ne renoncer a aucun droit legal a cause de votre participation a cette etude. Si vous avez des questions regardant votre droit en tant que participant a une etude de recherché vous pouvez contacter: IRB bureau de la FMPOS/Universite de Bamako au 222 7752 Universite de Bamako (MALI). BP: 1805 Tel 223 675 44 24

CONFIRMATION DE LA COMPREHENSION:

S'il s'agit d'une verbale plutôt que par accord écrit, s'il vous plaît nous expliquer ce que nous sommes d'accord, afin de confirmer qu'il n'y ait pas de malentendus.

SIGNATURE DE L'INVESTIGATEUR OU L'AUTORISE:

J'ai clairement expliqué la forme de consentement éclaire a un niveau approprie pour que le répondant puisse comprendre. Il (le répondant) a volontairement accepté de participe a cette étude.

Signature de l'enquêteur

______________________________________ Date__________________

J'ai vérifié que la traduction dans les langues suivantes ________________________était claire.

Signature du traducteur

____Date________________

University of California, Los Angeles

FICHE D'INFORMATION SUR LA RECHERCHE

Moustiques Genetiquement modifies pour la lutte contre le Paludisme:
Attitudes par rapport a la biotechnologie au Mali, Afrique de l'Ouest

Nous venons vous demander de bien vouloir participer a une etude menee par Dr Mahmoudou Toure, Dr John Marshall et Dr Charles Taylor du departement de biologie et d'ecologie de l'Universite de California a Los Angeles. Vous aviez ete selectionne pour participer a cette etude parceque les responsables scientifiques du Centre de Recherche et de Formation sur le paludisme (MRTC/FMPOS) de Bamako ont cru necessaires d'obtenir vos idees et conceptions sur les questions specifiques qui nous interessent. Votre participation est totalement volontaire.

OBJECTIVE DE L'ETUDE:

Obtenir un apercu general de l'attitude du people malien par rapport a la biotechnologie. Il s'agit de comprendre les concepts et les valeurs qui accompagnent cette attitude.

PROCEDURES

Si vous accepter de participer a notre etude, nous allons vous pose quelques questions:

Nous vous prions de repondre aux questions qui suivent. Vous pouvez lire les questions en anglais ou en francais et il en est de meme pour les reponses (cela est a votre choix). Mais si vous preferez repondre oralement, nous allons enregistrer vos reponses sur un Dictaphone.

Il est tres important d'etre honnete dans vos reponses a chaque question. Les informations recueillies peuvent etre soumises aux decideurs qui sont interesses par vos opinions. L'enquete peut prendre 30 a 45 mn et se fait seulement une fois et a votre lieu de preference. Mais si vous vous voulez ecrire vos reponses, vous avez la possibilite de remplir le questionnaire quand et ou vous voulez et nous les envoyer au MRTC/FMPOS.

Si vous etes confus par rapport au questionnaire, nous vous encourageons a nous les poser a tout moment. Aussi si vous ne voulez pas repondre a une question specifique, vous pouvez tout simplement continuer avec la suivante et laissez la place vide.

RISQUES POTENTIELS OU NON-CONFORTABLES:

Il n'y a aucun risqué direct ou psychologique par rapport a votre participation a l'etude. Au dela de tout ca, il est possible que vos collegues ou members de votre communite agissent differement s'ils decouvrent que vos opinions different des leurs. Nous vous rassurons qu'il y aura une confidentiallite totale en ce qui concerne vos reponses pendant et après l'etude.

BENEFICES:

Vous n'allez pas directement beneficie de votre participation a cette recherche.

Les resultants de cette etude peuvent contribuer a la comprehension des attitudes des populations du Mali par rapport a la biotechnologie. La biotechnologie est l'utilisation d'outils et de méthodes basées sur la biologie, e.g. nouveaux médicaments et vaccins. Un benefice potentiel serait l'utilisation des moustiques genetiquement modifies au Mali pour la lutte contre le paludisme. Vos reponses peuvent aider a eclairer l'acceptabilite de cette technologe.

REMUNERATION

Aucun participant ne serait payer pour sa participation a cette etude.

CONFIDENTIALITE

Cette enquete est totalement anonyme. Aucun identifiant ne serait associe aux informations obtenues. Les enregistrements seront seront accessibles aux investigateurs seuls. Une fois l'ettude terminee, ces enregistrements seront mis dans une armoire fermee a clef au MRTC, avec une copie numerique dans le bureau du principal ionvestigateur a UCLA. Elles seront encryptees et protégées par un mot de passe secret.

PARTICIPATION AND DESISTEMENT

Vous pouvez choisir de participer ou pas a cette etude. Si vous acceptiez volontier d'y participer, vous pouvez vous retirer a tout moment. Ce retrait est sans consequence pour vous.

IDENTIFICATION DES INVESTIGATEURS

Si vous avez une question concernant cette etude, nous vous prions de contacter le Dr Mahamoudou Toure (Tel: 223 6778912 ou email: mah.toure@gmail.com, addresse: MRTC, Faculty of Medicine, University of Bamako, Bamako, Mali), John Marshall (phone: 223-411-5334, email: john.macky.marshall@gmail.com, address: MRTC, Faculty of Medicine, University of Bamako, Bamako, Mali), Prof Charles Taylor (phone: +1-310-206-3987, address: email: taylor@biology.ucla.edu, address: Department of Ecology and Evolutionary Biology, University of California, Los Angeles, CA 90095, USA).

DROITS:

Vous pouvez vous retirer à tout moment et decider de re-participer sans aucune consequence. Vous ne renoncer a aucun droit legal a cause de votre participation a cette etude. Si vous avez des questions regardant votre droit en tant que participant a une etude de recherché vous pouvez contacter: IRB bureau de la FMPOS/Universite de Bamako au 222 7752 Universite de Bamako (MALI). BP: 1805 Tel 223 675 44 24

CONFIRMATION DE LA COMPREHENSION:

S'il s'agit d'une verbale plutôt que par accord écrit, s'il vous plaît nous expliquer ce que nous sommes d'accord, afin de confirmer qu'il n'y ait pas de malentendus.

SIGNATURE DE L'INVESTIGATEUR OU L'AUTORISE:

J'ai clairement expliqué la forme de consentement éclaire a un niveau approprie pour que le répondant puisse comprendre. Il (le répondant) a volontairement accepté de participe a cette étude.

Signature de l'enquêteur

____ Date___________________

J'ai vérifié que la traduction dans les langues suivantes _________________________ était claire.

Signature du traducteur

____Date_________________

Questions for a survey of rural areas in Mali on perspectives related to the use of genetically modified mosquitoes intended to control malaria:

<u>Demographic information:</u>

Age
Gender
Marital Status
Number of children
Level of education
Role in the community
Religion
Importance of religion

<u>Questions on the perception of disease:</u>

Malaria is a common disease in Africa with symptoms including fever, shivering, joint pain, vomiting, anemia and convulsions.

a: What do you think is the primary cause of malaria?
b: Have you or anyone in your family ever had malaria?
c: Has anyone in your family died as a result of a malaria infection?

<u>Questions on the sacredness of nature:</u>

Many cultures throughout the world have views on the sacredness of nature, and what alterations to the environment are acceptable or unacceptable.

a: What are your views on the intrinsic value of nature and the environment?
b: What environmental alterations are acceptable and unacceptable in your culture?
c: Can you think of any occasions when the values attributed to nature have conflicted with other values or ambitions in your community?

<u>Questions on cultural views towards living creatures:</u>

Some cultures view all living creatures as possessing a life energy or soul. Some cultures consider even inanimate objects such as cliffs, stones and water to possess this force.

a: What are the views of your culture have towards living creatures?
b: How do these views influence your respect towards animals and your treatment of them?

<u>Questions on genetics and heredity:</u>

In nature, it is common for offspring to resemble their parents.

a: What do you consider to be the reason for this resemblance?
b: Does your community take advantage of this resemblance to selectively raise the most desirable animals, vegetables or fruits?

<u>Questions on genetic alteration:</u>

Imagine that there was a faster way to develop more desirable animals, vegetables or fruits, but that this method could lead to unknown consequences for the environment.

a: In your opinion, would it be acceptable to raise these animals or grow these fruits and vegetables in your community?
b: What questions and concerns would you have regarding this new method?

<u>Questions on trust in foreign organizations:</u>

Imagine that an organization from a foreign country gifts you a more desirable fruit or vegetable that has been produced by this method; for example, a corn that is resistant to insects. A representative from the organization tells you that the crop has no negative consequences for the environment.

a: Under what circumstances would you trust the representative and his statement that there are no negative consequences for the environment?
b: If an international organization, such as the United Nations, said that the crop had no negative consequences for the environment, would you trust their opinion?
c: If the Malian government said that the crop had no negative consequences for the environment, would you trust their opinion?
d: Under what circumstances, if any, would you consider it acceptable to grow this vegetable in your community?

<u>Questions on genetic alteration for health:</u>

Now imagine that an organization from a foreign country claims that they can provide you with a mosquito that has been produced by this method which is able to reduce the burden of malaria in your community. A representative from the United Nations tells you that the mosquito has no known negative consequences for your community or the environment. They do tell you, however, that there could be possible unknown negative consequences.

a: How much trust would you have in the claim of the foreign organization that they can reduce the burden of malaria by releasing these mosquitoes?
b: What questions and concerns would you have regarding this project?
c: Under what conditions, if any, would you consider it acceptable for the foreign organization to release these mosquitoes into your community?

Questions on the decision-making process:

Cultures throughout the world have different approaches to linking their basic values with decision-making.

a: How are decisions arrived at in your community when there are implications for all community-members?
b: Who is involved in the decision-making process?
c: Is consensus important in this process?
d: How do you act upon a decision once it has been made?
e: Are decisions made by your community final, or is it possible to reconsider them at a later date?

Questions on decisions made on a larger geographical scale:

National governments, state governments and city and regional councils sometimes make decisions that have implications for local communities.

a: Have important decisions made by larger governing bodies ever conflicted with the wishes of your local community?
b: If so, how has your community dealt with this?
c: Are there realistic ways in which your community is able to contribute to decisions made on a larger scale?
d: When decisions are made on a national level that are against the wishes of your community, does your community tend to accept the consequences of this decision, or are there sometimes alternative courses of action?
e: Do you think that there is usually a way ahead that can take into account values shared by all communities?

Questions on the Western decision-making process:

Decisions are often made in Western societies by committees. These committees are usually composed of elected or co-opted members and meet at regular intervals. They run by an agenda, take detailed written records of all opinions, and are over in a few hours.

a: Are you familiar with the Western decision-making process?
b: How similar is your community's decision-making process to the Western process?

Questions for a survey of urban areas in Mali on perspectives related to the use of genetically modified mosquitoes intended to control malaria:

Demographic information:

Age
Gender
Marital Status
Number of children
Level of education
Occupation
Religion
Importance of religion

Questions on the perception of disease:

Malaria is a common disease in Africa with symptoms including fever, shivering, joint pain, vomiting, anemia and convulsions.

a: What do you think is the primary cause of malaria?
b: Have you or anyone in your family ever had malaria?
c: Has anyone in your family died as a result of a malaria infection?

Questions on the intrinsic value of nature:

Many people have views on the intrinsic value of nature, and what alterations to the environment are acceptable or unacceptable.

a: What are your views on the intrinsic value of nature and the environment?
b: What environmental alterations do you consider acceptable and unacceptable?

Questions on cultural views towards living creatures:

Some cultures view all living creatures as possessing a life energy or soul. Some cultures consider even inanimate objects such as cliffs, stones and water to possess this force.

a: What are your views towards living creatures?
b: How do these views influence your respect towards animals and your treatment of them?

Questions on genetics and heredity:

In nature, it is common for offspring to resemble their parents. What do you consider to be the reason for this resemblance?

Question on image of biotechnology:

What comes to mind when you think about modern biotechnology in a broad sense (including genetic engineering)?

Questions on specific applications of biotechnology:

a: For each of the specific applications of biotechnology that follow:

1. Genetically-modified food
2. Pest-resistant crops, for example, corn resistant to insects
3. Human genes in bacteria, for example, to produce insulin for diabetics
4. Pigs with human hearts for heart transplants
5. Mosquitoes unable to carry human disease

Please answer the following questions:

- Have you heard of this application? (Yes, No, Don't know)
- How useful do you think this application is for society? (Very useful, Useful, Useless, Very useless, Don't know)
- How risky do you think this application is for society? (Very risky, Risky, Safe, Very safe, Don't know)
- How morally acceptable do you think this application is? (Very acceptable, Acceptable, Unacceptable, Very unacceptable, Don't know)
- All in all, do you this application should be encouraged? (Definitely yes, Yes, No, Definitely no, Don't know)

b: If there was no direct risk to humans and only very remote risks to the environment, would you approve or disapprove of the environmental use of genetically modified organisms designed to produce:

1. Tomatoes with better taste? (Yes, No, Don't know)
2. Larger sport fish? (Yes, No, Don't know)
3. Bacteria to clean up oil spills? (Yes, No, Don't know)
4. Disease-resistant crops? (Yes, No, Don't know)
5. Cows which produce more milk? (Yes, No, Don't know)
6. Mosquitoes unable to carry human disease? (Yes, No, Don't know)

Questions on expectations of biotechnology:

Following is a list of things that might happen as a result of modern biotechnology. For each one, please state whether you think it is likely or unlikely to happen with the next 20 years:

1. Substantially reducing environmental pollution? (Likely, Unlikely, Don't know)
2. Substantially reducing world hunger? (Likely, Unlikely, Don't know)

3. Creating dangerous new diseases? (Likely, Unlikely, Don’t know)
4. Reducing the range of fruits and vegetables? (Likely, Unlikely, Don’t know)
5. Reducing the burden of disease throughout Africa? (Likely, Unlikely, Don’t know)

<u>Questions on risks, benefits and regulation of biotechnology:</u>

a: Following are a list of statements about the risks, benefits and regulation of modern biotechnology. For each one, please say whether you tend to agree or disagree.

1. Biotechnologists will do whatever they like irrespective of regulations. (Agree, Disagree, Don’t know)
2. Changing the hereditary characteristics of plants and animals through modern biotechnology is impermissible. Only traditional breeding methods should be used. (Agree, Disagree, Don’t know)
3. Modern biotechnology is so complex that public consultation about it is a waste of time. (Agree, Disagree, Don’t know)
4. Religious organizations need to have their say in how modern biotechnology is regulated. (Agree, Disagree, Don’t know)
5. We have to accept some degree of risk from modern biotechnology if it has potential to reduce the prevalence of disease. (Agree, Disagree, Don’t know)

b: Overall, do you think science and technology do more harm than good, more good than harm, or about the same of each? (More harm, More good, Same, Don’t know)

<u>Questions on trust in local and foreign organizations:</u>

a: Which of the following bodies do you think is best-placed to regulate modern biotechnology?

- United Nations organizations
- Government authorities
- Ethics committees
- Scientific organizations
- Others
- Don’t know

b: Which of the following sources of information do you have confidence in to tell you the truth about modern biotechnology?

- Consumer organizations
- Environmental organizations
- Animal welfare organizations
- Political parties
- Trade unions
- Religious organizations

- Public authorities
- Industry
- Schools and universities
- Media

Questions on the presence of debate on biotechnology in the Malian media:

Have you heard any stories about issues involving modern biotechnology in Mali over the last three months? If so:

a: What media did you hear this story from?
b: What was the story about?

Questions on the decision-making process:

a: Over what time frame do you think debates on complex scientific, cultural and spiritual matters should proceed?
b: Who do you think should be involved in this decision-making process?
c: How important to you think consensus is?
d: What steps do you think should be taken to ensure that opinions from isolated communities can be heard?
e: How do you think we can address the lack of technical expertise in local communities to deal with such issues?
f: How do you think cultural and spiritual values can be accounted for in the decision-making process without simply being dismissed?
g: Do you think there is a common core of values throughout Mali, and it so, what are these values?

Questions on the Western decision-making process:

Decisions are often made in Western societies by committees. These committees are usually composed of elected or co-opted members and meet at regular intervals. They run by an agenda, take detailed written records of all opinions, and are over in a few hours.

a: Are you familiar with the Western decision-making process?
b: How similar is your community's decision-making process to the Western process?

Questions for a survey of scientists and medical professionals in Mali on perspectives related to the use of genetically modified mosquitoes intended to control malaria:

Demographic information:

Age
Gender
Marital Status
Number of children
Level of education
Occupation
Religion
Importance of religion

Questions on the perception of disease:

Malaria is a common disease in Africa with symptoms including fever, shivering, joint pain, vomiting, anemia and convulsions.

a: What do you think is the primary cause of malaria?
b: Have you or anyone in your family ever had malaria?
c: Has anyone in your family died as a result of a malaria infection?

Questions on the intrinsic value of nature:

Many people have views on the intrinsic value of nature, and what alterations to the environment are acceptable or unacceptable.

a: What are your views on the intrinsic value of nature and the environment?
b: What environmental alterations do you consider acceptable and unacceptable?

Questions on cultural views towards living creatures:

Some cultures view all living creatures as possessing a life energy or soul. Some cultures consider even inanimate objects such as cliffs, stones and water to possess this force.

a: What are your views towards living creatures?
b: How do these views influence your respect towards animals and your treatment of them?

Question on image of biotechnology:

What comes to mind when you think about modern biotechnology in a broad sense (including genetic engineering)?

Question on interspecies gene transfer:

Genes from most types of organisms are interchangeable. Would rice made more nutritious through biotechnology be acceptable:

a: If genes were added from another type of plant, such as corn? (Yes, No, Don’t know)
b: If the new genes came from an animal, such as a horse? (Yes, No, Don’t know)

Questions on specific applications of biotechnology:

a: For each of the specific applications of biotechnology that follow:

6. Genetically-modified food
7. Pest-resistant crops, for example, corn resistant to insects
8. Human genes in bacteria, for example, to produce insulin for diabetics
9. Pigs with human hearts for heart transplants
10. Mosquitoes unable to carry human disease

Please answer the following questions:

- Have you heard of this application? (Yes, No, Don’t know)
- How useful do you think this application is for society? (Very useful, Useful, Useless, Very useless, Don’t know)
- How risky do you think this application is for society? (Very risky, Risky, Safe, Very safe, Don’t know)
- How morally acceptable do you think this application is? (Very acceptable, Acceptable, Unacceptable, Very unacceptable, Don’t know)
- All in all, do you this application should be encouraged? (Definitely yes, Yes, No, Definitely no, Don’t know)

b: If there was no direct risk to humans and only very remote risks to the environment, would you approve or disapprove of the environmental use of genetically modified organisms designed to produce:

7. Tomatoes with better taste? (Yes, No, Don’t know)
8. Larger sport fish? (Yes, No, Don’t know)
9. Bacteria to clean up oil spills? (Yes, No, Don’t know)
10. Disease-resistant crops? (Yes, No, Don’t know)
11. Cows which produce more milk? (Yes, No, Don’t know)
12. Mosquitoes unable to carry human disease? (Yes, No, Don’t know)

Questions on expectations of biotechnology:

Following is a list of things that might happen as a result of modern biotechnology. For each one, please state whether you think it is likely or unlikely to happen with the next 20 years:

6. Substantially reducing environmental pollution? (Likely, Unlikely, Don’t know)
7. Substantially reducing world hunger? (Likely, Unlikely, Don’t know)
8. Creating dangerous new diseases? (Likely, Unlikely, Don’t know)
9. Reducing the range of fruits and vegetables? (Likely, Unlikely, Don’t know)
10. Reducing the burden of disease throughout Africa? (Likely, Unlikely, Don’t know)

Questions on risks, benefits and regulation of biotechnology:

a: Following are a list of statements about the risks, benefits and regulation of modern biotechnology. For each one, please say whether you tend to agree or disagree.

6. Biotechnologists will do whatever they like irrespective of regulations. (Agree, Disagree, Don’t know)
7. Changing the hereditary characteristics of plants and animals through modern biotechnology is impermissible. Only traditional breeding methods should be used. (Agree, Disagree, Don’t know)
8. Modern biotechnology is so complex that public consultation about it is a waste of time. (Agree, Disagree, Don’t know)
9. Religious organizations need to have their say in how modern biotechnology is regulated. (Agree, Disagree, Don’t know)
10. We have to accept some degree of risk from modern biotechnology if it has potential to reduce the prevalence of disease. (Agree, Disagree, Don’t know)

b: Overall, do you think science and technology do more harm than good, more good than harm, or about the same of each? (More harm, More good, Same, Don’t know)

Questions on trust in local and foreign organizations:

a: Which of the following bodies do you think is best-placed to regulate modern biotechnology?

- United Nations organizations
- Government authorities
- Ethics committees
- Scientific organizations
- Others
- Don’t know

b: Which of the following sources of information do you have confidence in to tell you the truth about modern biotechnology?

- Consumer organizations
- Environmental organizations
- Animal welfare organizations

- Political parties
- Trade unions
- Religious organizations
- Public authorities
- Industry
- Schools and universities
- Media

<u>Questions on the presence of debate on biotechnology in the Malian media:</u>

Have you heard any stories about issues involving modern biotechnology in Mali over the last three months? If so:

a: What media did you hear this story from?
b: What was the story about?

<u>Questions on the decision-making process:</u>

a: Over what time frame do you think debates on complex scientific, cultural and spiritual matters should proceed?
b: Who do you think should be involved in this decision-making process?
c: How important to you think consensus is?
d: What steps do you think should be taken to ensure that opinions from isolated communities can be heard?
e: How do you think we can address the lack of technical expertise in local communities to deal with such issues?
f: How do you think cultural and spiritual values can be accounted for in the decision-making process without simply being dismissed?
g: Do you think there is a common core of values throughout Mali, and it so, what are these values?
