# Supplementary material for: Perspectives of people in Mali toward genetically-modified mosquitoes for malaria control
Source: Malar J. 2010 May 14;9:128. doi: 10.1186/1475-2875-9-128 (PMC2881074; doi:10.1186/1475-2875-9-128)
Supplement: Additional file 3 — Necessary conditions for a release of GM corn cited by rural and urban populations, doctors, scientists and traditional healers. [file 1475-2875-9-128-S3.DOC]

**Additional file 3 - Table - Conditions for a release of GM crops**

|  | Conditions for a release of GM corn |
| --- | --- |
| Rural areas | Trial to confirm safety and efficacy (usually in own village, sometimes in similar environment)  Experiments conducted by Malian Ministry of Agriculture to confirm safety and efficacy  Access to detailed information about crop  Assurance from Malian government, Malian institution, local agricultural specialists, United Nations, foreign exporter  Dialogue between community and scientists  Approval by majority of community  Affordable for community |
| Urban areas | Evidence that GM corn will not cause human health concerns, environmental concerns  Trial to confirm safety and efficacy  Corn able to be sustainably grown for generations  Ability to preserve excess yields  Does not require many chemicals  Foreign scientists work with African scientists  GM corn used in moderation  Maintain a stock of traditional corn in case there are unforeseen problems |
| Doctors & scientists | Laboratory experiments conducted by foreign exporter to confirm safety  Evidence that GM corn produces a good yield in the local environment and will not cause environmental concerns  Assurance from Malian government, United Nations |
| Traditional healers | Trial to confirm safety and efficacy (in own village)  Evidence that GM corn is adapted to local environment  Assurance from Malian government, United Nations, foreign exporter  Foreign exporter has already conducted other projects in the community  Approval by majority of community  Ability to abort project  Education campaign by foreign exporter and tools provided so that local farmers can grow GM corn independently  Maintain a stock of traditional corn in case there are unforeseen problems |
