# Supplementary material for: Perspectives of people in Mali toward genetically-modified mosquitoes for malaria control
Source: Malar J. 2010 May 14;9:128. doi: 10.1186/1475-2875-9-128 (PMC2881074; doi:10.1186/1475-2875-9-128)
Supplement: Additional file 4 — Most trusted organizations for issues related to biotechnology among rural and urban populations, doctors, scientists and traditional healers. [file 1475-2875-9-128-S4.DOC]

**Additional file 4 - Table - Trust in local and foreign organizations**

| Trusted organizations for issues related to biotechnology | Rural areas^*^ | Urban areas^*^ | Doctors & scientists^*^ | Traditional healers^*^ | Total^*^ |
| --- | --- | --- | --- | --- | --- |
| Malian government, Malian institutions | 22 | 10 | 5 | 5 | 42 |
| United Nations, World Health Organization | 17 | 6 | 5 | 5 | 33 |
| Foreign exporter | 13 | 3 | 3 | 3 | 22 |
| Scientific organizations | – | 15 | 5 | – | 20 |
| Local agricultural specialists, local medical staff | 9 | 2 | – | 1 | 12 |
| Ethics committees | – | 3 | 6 | – | 9 |
| Schools, religious bodies, universities | – | 3 | – | – | 3 |

^*^ Number of interviewees
